# Supplementary material for: Experiences and challenges of parents caring for children with attention-deficit hyperactivity disorder: A qualitative study in Dar es salaam, Tanzania
Source: PLoS One. 2022 Aug 3;17(8):e0267773. doi: 10.1371/journal.pone.0267773 (PMC9348639; doi:10.1371/journal.pone.0267773)
Supplement: S1 Data — (DOCX) [file pone.0267773.s001.docx]

**Experiences and challenges of parents caring for children with attention-deficit hyperactivity disorder: A qualitative study in Dar es salaam, Tanzania**

**Sociodemographic characteristics data**

| Characteristic | Type | Frequency/Number |
| --- | --- | --- |
| Age | Between 30 and 39 | 5 |
|  | 40 years and above | 9 |
| Type of parents | Biological fathers | 4 |
|  | Biological mothers | 12 |
| Level of Education | Primary education,. | 8 |
|  | Secondary education, | 5 |
|  | College education | 3 |
| Marital Status | Single | 2 |
|  | Married | 10 |
|  | Divorced | 4 |
| Employment Status | Employed | 5 |
|  | Self-employed | 10 |
|  | Not employed | 1 |
| Residence | Urban | 6 |
|  | Rural | 10 |

**Excerpts of the transcripts**

**Challenges in handling child’s abnormal behaviour**

**Child’s Safety concern**

*“Last week …I was here (hospital) with him, he fell in the well which had no water he sustained many injuries and fracture on his left foot…. I am confused doctor” (Female,49 years old)*

*“Doctor this child…I cannot go to public places or use Daladala (commuter bus) together with him; he grabs items from people and sometimes hits people who try to warn him with stones. He should remain locked inside a room for the whole day”. (Female, 45-years-old,)*

“*I always use sticks beating him but this child is not listening and does not seem to change My wife is struggling and puts a lot of effort to change and makes him like other children…She does a lot”* (*Male, 40 years old*).

**Parental reaction to child’ disruptive behaviour**

“…*He is using the medications … but he is not calm (..) jumping here and there…I don't know what to do…breaking windows or anything…. It is terrible"… (Female,49years old).*

“...*One day my husband was beating the child too terrible and I was shocked (...) when I tried to stop him, he turned on to me. Always beating the child but it does not help” (Female,49 years old).*

*“…He is running a lot, jumping from one place to another he is always on the move even when we take food it makes us mad and sometimes I take him out and give him some work to do for his energy”. (Female,42years old)*

*“He cannot play for more than ten minutes, after a few minutes you will be called that there is a big fight and he beats other children then… I go to rescue and he will start to fight me back” (Female,38years old)*

**Psychological problems associated with caring demands**

**Lack of emotional support**

*“Look at me… Doctor this is not my body I was having a very healthy body everything has changed in my life…. my hair, my skin, my clothes ……My God …sometimes people think that I was being admitted or I am seriously sick” (Female,37years old).*

“…*Yah, I have to feel that way (pretending okay) even if I feel bad, but the child is already mine I cannot leave to someone else. I have to accept the way it is and wait for God to help in the care..." (Female,42years old).*

“*Sometimes I feel so alone and would like to chat with other parents about how they handle it all, it can all be so hard. I think that would also be helpful to hear […] from the other parents as well...”. (Female,38years old)*

**Social discrimination**

*“I don't feel worth living, I am socially isolated and lack support from my relatives, friends, and health professionals. I think there is ‘no use to tell others to get help or advice because they don’t understand me”.* (*F,30years old*)

*“We may benefit from face-to-face peer support or Web-based support groups, social networks, and social media, if we are connected to the world we could get assistance from the globe.”* (*Male, 40years old*)

*“Last year there was an election to our savings and credit cooperative society, I decided to take part to be chosen as one of the committee member, but they did not accept my name as some of the members thought that I am also sick and my child has inherited ADHD from me ...thus I am not fit to be a leader”. (Female,38years old)*

**Family and social dysfunctions**

**Disrupted of family process**

*“I’m a single mother with two children ... one is okay but this one only God knows…their father is enjoying life there... we divorced two years ago and he does not care or even visit us.” (Female,37years old)*

*“Disruptive behaviours of my child caused him to have academic underperformance, disciplinary issues at home and school. ﻿This affects all areas of family life such as relationships in the family and relatives*”. (*Female,30years old*)

**Disrupted neighbourhood relationship**

*“I ...remember when we were living at Gongolamboto* (a street in Dar es Salaam*),.. the landlord expelled us and gave back the money we paid for six months because of the child’s behaviours of breaking the windows and doors.” (Female,54years old)*

*“...When we get visitors at home; they will not stay long …I remember one day I attended a party at my neighbour’s house, what he did is horrible …beating other children... taking off clothes, threatening to beat other children around…. I decided to leave immediately” (Male,32 years old)*

“*We have no support from our neighbours, always blaming us as bad parents, that we are not responsible parents…. they don’t understand* *the nature of ADHD and its effects…. We are living in our own”* (*Male, 40years old*).

﻿

*“Last year we had a case to the police station with one of the neighbours after long term conflict with them, they demanded that we should lock inside our child, after breaking their 40 inch flat-screen television. It’s too expensive for us to pay but they still insist that we should pay back their money or buy the flat-screen television.”* (*Male, 40years old*)

**Disrupted family economic activities**

**Lack of household manpower**

“…. *I rush back to help my wife because there was no house girl to stay with the child. All house girls ran away even despite that we promised to pay them a good amount of money!! They could not tolerate the child's stubbornness. My wife was expelled from her job.” (Male,47years old).*

*“I remember one election, in the village they needed some people to supervise the election, but because I did not know anyone I missed the chance that I believed it could have helped me, others said they did not choose me because they know I have a sick child at home”* (*Female,30years old*). ﻿

“*I used to have a vegetable garden where… I worked before but this child needs constant supervision, having a child with ADHD is a 24/7hour job. We don’t trust anyone even the siblings they always abuse the child so most of the time we stay at home” (Male,32years old)*

**Lack of financial support**

*“We had one house and one big pilot, but after this disaster (ADHD) we sold all of them, and we have rent a house we hoped that he will be cured but he still using medications. We were all working, but my wife stopped working to take care of our child”, (Male,47years old).*

*Our child is being expelled from school after we failed to pay school fees, my husband is trying a lot but we have remained poor since then …what we get all goes to manage our child” (Female,54years old).*

*“It is very expensive … the medication prices are horrible …. My biggest worry is he will be taking these medications for life with no health insurance” …. (Female,38years old).*

*“We are living outside this region the fare is also very costly … we need to travel every three months to attend the multiple clinics, the doctors think that we are not responsible but we are poor and not employed” (Male, 40years old).*
