# Supplementary material for: Experiences and challenges of parents caring for children with attention-deficit hyperactivity disorder: A qualitative study in Dar es salaam, Tanzania
Source: PLoS One. 2022 Aug 3;17(8):e0267773. doi: 10.1371/journal.pone.0267773 (PMC9348639; doi:10.1371/journal.pone.0267773)
Supplement: Attachment — Submitted filename: Response to Reviwers.docx [file pone.0267773.s002.docx]

**“Experiences and challenges of parents caring for children with attention-deficit hyperactivity disorder: A qualitative study in Dar es salaam, Tanzania”**

**RESPONSE TO REVIEWRS COMMENTS**

With many appreciations, we thank the reviewers for the comments they have provided meant to improve our study. We have done an extensive manuscript revision with our point-to-point responses to reviewers' comments provided in the table below. Responses provided can be traced in the uploaded manuscript's document named ‘Revised manuscript with track changes’. If any of our responses fail to address your comments, please let us know so that we can provide further details.

| **REVIWER’S COMMENTS** | **AUTHORS’ RESPONSE** |
| --- | --- |
| **Academic Editor’s Comments** |  |
| 1. Please ensure that your manuscript meets PLOS ONE's style requirements, including those for file naming. The PLOS ONE style templates can be found at  https://journals.plos.org/plosone/s/file?id=wjVg/PLOSOne_formatting_sample_main_body.pdf and  https://journals.plos.org/plosone/s/file?id=ba62/PLOSOne_formatting_sample_title_authors_affiliations.pdf | We have ensured that our manuscript follow PLOS ONE's style requirements by reading and adhering to the templates found at  https://journals.plos.org/plosone/s/file?id=wjVg/PLOSOne_formatting_sample_main_body.pdf and  https://journals.plos.org/plosone/s/file?id=ba62/PLOSOne_formatting_sample_title_authors_affiliations.pdf  For this reason, we have also deleted the section named ‘Implication for practice’ as it is not supported by the sections required. |
| 2. In your Data Availability statement, you have not specified where the minimal data set underlying the results described in your manuscript can be found. PLOS defines a study's minimal data set as the underlying data used to reach the conclusions drawn in the manuscript and any additional data required to replicate the reported study findings in their entirety. All PLOS journals require that the minimal data set be made fully available. For more information about our data policy, please see http://journals.plos.org/plosone/s/data-availability. | We appreciate the comment. We have made excerpts of the transcripts relevant to the study available by uploading them as Supporting Information files. |
| Upon re-submitting your revised manuscript, please upload your study’s minimal underlying data set as either Supporting Information files or to a stable, public repository and include the relevant URLs, DOIs, or accession numbers within your revised cover letter. For a list of acceptable repositories, please see http://journals.plos.org/plosone/s/data-availability#loc-recommended-repositories. Any potentially identifying patient information must be fully anonymized.  Important: If there are ethical or legal restrictions to sharing your data publicly, please explain these restrictions in detail. Please see our guidelines for more information on what we consider unacceptable restrictions to publicly sharing data: http://journals.plos.org/plosone/s/data-availability#loc-unacceptable-data-access-restrictions. Note that it is not acceptable for the authors to be the sole named individuals responsible for ensuring data access.  We will update your Data Availability statement to reflect the information you provide in your cover letter. | We appreciate the comment. We have uploaded our study’s minimal underlying data set as Supporting Information files. |
| 3. Please review your reference list to ensure that it is complete and correct. If you have cited papers that have been retracted, please include the rationale for doing so in the manuscript text, or remove these references and replace them with relevant current references. Any changes to the reference list should be mentioned in the rebuttal letter that accompanies your revised manuscript. If you need to cite a retracted article, indicate the article’s retracted status in the References list and also include a citation and full reference for the retraction notice. | We appreciate the comment. We have reviewed our reference list to ensure that it is complete and correct. We have also referred to the PLOSONE guidelines for preparing references to ensure that we meet the requirement. We have also added references number 20, 21, 22, and 23 due to information added in the process of addressing the reviewers’ comments |
| **Reviewer #1: Minor Revision;** |  |
| The Manuscript “Experiences and challenges of parents caring for children with attention-deficit hyperactivity disorder: A qualitative study in Dar es salaam, Tanzania” explore how parents struggle to meet and cope with care demands posed by children with ADHD.  It has got significance for the public health practices. | We appreciate the comments |
| Please mention the study approach i.e., Phenomenology. | We accept the comment. The study approach has been mentioned as phenomenology. |
| Provide the Interview guide in Tabular form. | We accept the comment. The interview guide has been modified into a tabular form. |
| How many participants were initially contacted and how many did not show their willingness to participate? | Thank you for the comment. We accept it and have added a summary of this information in the methods section under subsection ‘Selection of participants’ |
| Where the interviews were carried out? | The interviews were carried in a room that was available at Child and Adolescent Psychiatric clinic which was temporarily prepared to be used for conducting interviews. This information was provided in the Materials and methods section under sub-section ‘Data collection methods and tools’. However, information about this venue for interviews has been further revised for more clarity. |
| Instead of Study Limitations, write about methodological consideration with both strength and limitations. | We appreciate this good comment and accept it. We have removed Study Limitations section and included methodological consideration (with both strength and limitations) at the end of the discussion section. |
| Reduce quotation in the Results Section and describe it in the content of the results. | We appreciate the comment provided. Quotations in the Results section have been reduced by deleting those which were not necessary to reduce bulkiness and the summary of information carried by deleted quotes have been included in the content of the results. |
|  |  |
| **Reviewer #2:** |  |
| In title- the type of study (qualitative study) and place of study (Tanzania) need to be added. | We appreciate the comment. However, our titles reads “Experiences and challenges of parents caring for children with attention-deficit hyperactivity disorder: A qualitative study in Dar es salaam, Tanzania” which already contains the type and place of the study. |
| Sample size is low (only 16 IDI), FGD can be incorporated. | We appreciate this comment. However, we understand that in qualitative studies sample size is not the main issue. What is more important in qualitative studies is reaching ‘saturation’ with the information gathered i.e. when no new information is coming up as you continue to gather data. For this reason, we reached saturation at the 16^th^ in-depth interview. Moreover, we did not use FGDs because we wanted to get more deeper and varying personal experiences of parents as they cared for children with ADHD which we thought could better be achieved through in-depth interviews. |
| Description of content analysis need to be added with reference | Description of content analysis has been added including steps involved in the process. We have also added references to support the description. |
| Gender based findings of the parents and their children with ADHD can be compared | We appreciate the comment. However, qualitatively, we were much more interested in describing experiences and challenges of parents. We think that gender based findings of the parents and their children with ADHD could be better compared in a quantitative study. |
| Gender of the children with ADHD and gender based segregation of parental support can be added | We accept the comment. We have added age and gender of the children in table 3 (sociodemographic characteristics of participants). Gender based segregation of parental support is reflected in the sociodemographic data. However, since we did not explore this we have included it in the discussion section as a limitation to our study. |
| Analytical findings can be expressed in percentages also (eg- how many/% of them faced challenges) | We appreciated this comment. All interviewed participants faced challenges one way or the other. In qualitative studies emphasis or interest is more placed on the quality (insight and understanding of phenomena through intensive collection of narrative data rather than quantity such as percentages or frequencies). We are of the opinion that this comment could be better addressed if this was a quantitative study. |
